# Monkeypox: A comprehensive review with a focus on the Middle East and North Africa (MENA) region

**DOI:** 10.5339/qmj.2025.52

**Published:** 2025-06-18

**Authors:** Aya A. Al-Rubaye

**Affiliations:** 1 Basrah Medical College, Basrah, Iraq *Email: aya.zayed@uobasrah.edu.iq

**Keywords:** Mpox, monkeypox, public health, epidemiology

## Abstract

**Background:** Monkeypox (Mpox) was identified as a virus endemic to Central and Western Africa. Since 2022, the virus has gained global attention due to increasing cases in non-endemic countries among patients having no link to travel to endemic areas. The virus is primarily transmitted through animal-to-human contact but has increasingly spread via human-to-human transmission. A comprehensive understanding of the epidemiology of Mpox in the Middle East and North Africa (MENA) region is essential for effective disease prevention, diagnosis, surveillance, and control.

**Methods:** This review investigates historical data and recent global and regional epidemiological trends of Mpox. The review discusses the clinical features, public health challenges, and preventive measures relevant to the MENA region using updated data from World Health Organization (WHO) reports, national health statistics, and additional relevant resources.

**Results:** Mpox cases significantly spread globally during the 2022–2024 period. The MENA region has a relatively low number of documented cases, with 857 confirmed cases by August 2024. Saudi Arabia and the United Arab Emirates (UAE) reported the highest case numbers, reflecting effective surveillance and case detection. However, new cases of Mpox were announced in September 2024 in Morocco and Jordan following the WHO’s declaration of Mpox as a public health emergency of international concern.

**Conclusion:** The current number of cases in the MENA region could be underestimated due to underreporting, stigmatization, limited resources, and ongoing conflicts in several countries. It is essential to prioritize the containment of the outbreak, exploring all possible strategies to protect vulnerable communities.

## BACKGROUND

### Historical overview

Monkeypox virus (MPXV) was first identified in 1958 as a presentation of a pox-like illness in captive monkeys at the State Serum Institute in Copenhagen, from which the name “monkeypox” (Mpox) came.^1^ The term “monkeypox” is misleading because rodents were recognized as the major animal reservoirs of the virus.^2^

The first recognized human infection in medical history was detected in 1970 in Congo. The case involved a 9-month-old infant initially suspected of having smallpox, but—after its eradication—was later diagnosed with Mpox. The condition was clinically indistinguishable from smallpox infection.^3^ The extent of wild animal reservoirs, as well as the natural history and pathogenesis of Mpox in animals and humans, remains largely unclear.^2^ The virus is endemic to Central and Western Africa.^4^ Human Mpox cases have steadily increased since its discovery. This was thought to be caused by the lack of immunity to orthopoxviruses after the discontinuation of mass smallpox vaccination campaigns in the early 1980s, which led to increased numbers of susceptible individuals, especially among younger ages.^5^ From 1981 to 1986, the World Health Organization (WHO) implemented an active surveillance program for human Mpox. This initiative aimed to detect and monitor Mpox cases in the Democratic Republic of the Congo (DRC) in which 338 out of the 404 total cases in Africa were documented. Since the discontinuation of the WHO surveillance program, it has been suggested that the number of Mpox cases in endemic regions is greater than what has been documented in published articles.^6^ Sporadic cases were reported, and an outbreak was documented in 1997 in endemic regions.^7^ The situation remained unchanged until the Centers for Disease Control (CDC) announced an outbreak of Mpox in the United States in June 2003.^8,9^ The source of this outbreak was traced to native prairie dogs (genus Cynomys) kept with exotic pets imported from Africa.^10^ Close contact between humans and infected pets resulted in 37 confirmed human infections by January 2004, most presenting as mild, self-limited febrile rash illnesses.^11^ Transmission was primarily by large droplets or direct contact with the pets.^10^ Two pediatric patients had significant complications that necessitated hospitalization; one had severe encephalitis, and the other had severely painful cervical and tonsillar lymphadenopathy. This incident represented the first reported case of Mpox in the Western world.^6^

## CAUSATIVE AGENT

MPXV is an Orthopoxvirus, genetically distinct from other viruses in the Poxviridae family.^4^ The MPXV is one of four Orthopoxvirus species that cause disease in humans. This group includes the Variola virus (smallpox), Cowpox virus, and Vaccinia virus.^12^ Mpox is a double-stranded DNA virus primarily found in rodents and other animals. It is believed to have been transmitted to humans through incidental contact with infected animals and gained the ability to spread further via human-to-human transmission.

There are two distinct clades of MPXV: clade I, which was previously known as the Congo Basin clade or Central African clade; and clade II, which was called the West African clade. Clade II of the MPXV is divided into two subclades, IIa and IIb. Subclade IIb was responsible for the global outbreak that occurred in 2022.^13,14^

Clade I infections are associated with more severe presentation, raised case-fatality ratios, and demonstrated higher rates of human-to-human transmission compared to clade II.^15^

## MODE OF TRANSMISSION

For the primary transmission from animal to human, e.g. zoonotic transmission, the virus enters from the bodily fluids of infected animals via contact with skin lesions or mucous membranes or respiratory transmission.^16,17^ Risk factors include exposure to animal reservoirs, particularly in areas where increased animal-human interaction exists, as well as handling or consuming undercooked meat from infected animals.^18^

Although the human-to-human mode of transmission of the virus was confirmed in research, the exact mechanism is still largely unclear.^16^ The transmission is likely to occur through extended close contact with an infected individual’s skin lesions, respiratory secretions, or contaminated surfaces.^16,19^ Vertical mother-to-child transmission was also documented.^20^ MPXV was detected in the semen, with higher rates of infection in the 2022 outbreak among bisexual, men having sex with men, and gay populations indicating sexual transmission.^18^

## CLINICAL FEATURES

Mpox is usually a self-limiting disease. The symptoms last for 2–4 weeks in most of the cases.^21^ The incubation period ranges from 5 to 21 days with an average of 6–13 days. Mpox typically presents with a prodrome of flu-like symptoms including fever, backache, malaise, sore throat, and headaches.^22^ Lymphadenopathy is observed in many patients 1–2 days before the onset of the rash and is a key distinguishing feature of human smallpox.^4^ Approximately 95% of Mpox patients experience fever resolution within 3 days, coinciding with a distinct rash that progresses from macules to pustules. This rash typically spreads centrally, affecting the face, palms, and soles.^22,23^ However, recent outbreaks have shown atypical clinical presentations, particularly among immunocompromised individuals. Thus, the diagnosis might be delayed, increasing the risk of transmission due to delayed isolation.^24^

However, a growing number of atypical clinical manifestations of Mpox since 2022 have been noticed. These include the presence of only a few or a single lesion. Other symptoms such as anal pain, bleeding, tenesmus, or diarrhea and lesions localized to the genital or perineal and perianal areas without further spread. Moreover, lesions might appear at different stages of development, and the prodromal period can be asymptomatic or unnoticed.^25,26^ Complications of Mpox include pneumonitis, sight-threatening keratitis, encephalitis, secondary bacterial infection, and sepsis.^21^

## MPOX IN RECENT YEARS

In September 2018, three cases of Mpox infection were reported in the United Kingdom (UK). Two of the patients had recently traveled to Nigeria, while the third was a healthcare provider who had come into contact with one of the infected patients. This incident confirmed human-to-human transmission of MPXV.^27^

On May 7, 2022, the WHO was notified of a confirmed Mpox case in a patient who had traveled from the UK to Nigeria and then returned to the UK. The case was promptly isolated due to suspicion of Mpox. Extensive contact tracing was conducted to identify exposed individuals in healthcare settings, the community, and during the international flight. However, the source of the infection in Nigeria remained unidentified.^28^ From May to June 2022, cases continued to be reported to the WHO from non-endemic countries, with patients having no history of travel to endemic countries.^29^ The 2022 outbreak resulted in 3413 laboratory-confirmed Mpox cases and the death of one case reported to the WHO. The number of cases is thought to be underestimated due to limited screening resources in several countries and the atypical/asymptomatic presentation of Mpox infection.^30^ The unique feature of this outbreak was that initial cases were mainly documented among men who had sex with men.^29^ Many cases did not present the classic symptoms. Common signs include genital and perianal lesions, fever, lymphadenopathy, and sore throat. The rash often starts locally in the anogenital area, suggesting close sexual contact as a primary transmission route. Hospitalizations were primarily for isolation, pain management, and treatment of secondary infections.^30^ This outbreak was the first instance of Mpox cases being reported simultaneously across multiple countries and continents, with no known epidemiological connections to endemic areas in West or Central Africa. The European Region accounted for 86% of the cases, followed by the African Region. The Eastern Mediterranean Region, which includes the Middle East and North Africa (MENA) countries, accounted for less than 1% of the reported cases during the 2022 outbreak.^30^ The WHO announced an estimated moderate risk of Mpox globally except for the European Region where the risk was classified as high.^31^ In May 2023, the WHO declared that the outbreak had been brought under control and the risk of further international spread had significantly reduced and was no longer considered a public health emergency of international concern (PHEIC).^32^

New cases, however, continue to emerge outside regions with previously recognized endemic transmission. In August 2024, an Emergency Committee was convened under the International Health Regulations to assess the increase in Mpox cases in the DRC and other African nations. On August 14, 2024, the committee determined that the situation qualifies as a public health PHEIC.^33^ Mpox has regained global attention as both the WHO and Africa CDC declare it a public health emergency. The DRC remains the epicenter of the outbreak, but the virus’s spread beyond Africa has caused significant international concern, particularly with the emergence of a new sexually transmissible strain of the MPXV named clade 1b.^34^

Clade 1b of Mpox is particularly worrisome due to its higher transmission rate and the potential for more severe health outcomes. According to an Africa CDC epidemic report from 31 August 2024, there have been 5265 confirmed and 18,737 suspected Mpox cases across 13 African nations since the beginning of the year, leading to 617 deaths with a case-fatality ratio of 2.57%.^35^ Globally, from January 1, 2022, until August 31, 2024, cases of Mpox have been reported to WHO, reaching a total of 106,310 laboratory-confirmed cases and 234 deaths, with a 15.6% increase in newly reported cases compared to reports from July 2024. The majority of these recent cases came from the African Region (62.3%) and the European Region (13.7%).^36^

Unlike the 2022 Mpox outbreak, which predominantly affected men who had sex with men, the 2024 outbreak is impacting a broader demographic. The virus primarily spreads through direct contact and has been reported among men, women, and children.^34^

## MPOX IN THE MENA REGION

There are limited published articles on Mpox spread in the MENA region. Most of the information about Mpox infections is derived from WHO statistics and official authorities of these countries and, in most instances, without giving further epidemiological details and characteristics of these cases.

The UAE was the first Arabian Gulf country to detect a Mpox case on May 24, 2022. The patient was a 29-year-old female who came on a visit from a West African country.^29,37^ Morocco was the second Arab country to announce a confirmed Mpox case by June 2022 in a patient who had a history of travel to Europe.^17,38^

According to the WHO Mpox global trends report updated data from 01 January 2024 to 31 August 2024, the total number of confirmed Mpox infections in Arab countries was 857 cases out of 106,310 cases documented globally (<1%). The mortality rate was low, with only one death recorded in Sudan.^36^

The highest number of cumulative cases since 2022 was reported in Saudi Arabia, with 764 patients, followed by the UAE with 28 laboratory-confirmed cases.^36^ In 2024 alone, Saudi Arabia reported 94 cases, while the UAE reported 3 cases.^38^ The elevated numbers in these two countries may be attributed to their effective case detection and monitoring systems and contact-tracing strategies. Furthermore, both countries have high levels of international travel for tourism and religious purposes and a high percentage of non-national residents. Moreover, they have well-structured healthcare facilities that are well-prepared to identify and manage health emergencies.^39^

A study by A.M. Assiri, et al. provided a detailed epidemiological profile of 381 Mpox cases in Saudi Arabia during the period from May to September 2023.^40^ The cases did not differ from the international outbreak in terms of age, sex, and clinical presentation. The majority of cases were young males who presented mainly with fever, headaches, and rash. However, 93.4% of them had no link to any travel history. The cases showed a predominance of subclade IIb.^40^ Therefore, the Mpox cases in Saudi Arabia are consistent with the current global outbreak, which has been driven by multiple subclades within the clade IIb lineage.^41^

Lebanon is ranked third among Arab countries in terms of cumulative Mpox cases with 27 documented cases between September 2022 and March 2023.^36^ However, no new cases have been reported since then.^42^ Similarly, Egypt, Qatar, Oman, and Bahrain reported no new cases since 2023 with a total of twenty cumulative cases in these countries.^38,43–46^ A study was conducted by Zaqout et al. in Qatar, who investigated twelve male cases of Mpox. Cases were reported between May and November 2022 with a history of being involved in unsafe sexual practices and anogenital rash distribution in most of the patients.^47^

Morocco and Jordan were the only countries in the MENA region to detect new cases of Mpox after the WHO’s new declaration of Mpox as a PHEIC on 14 August 2024.^32^ Jordan reported a new Mpox case in a non-national resident in September 2024, bringing the cumulative total to two cases since 2022.^36,48^ Morocco was another country that announced a new case in September, reaching a cumulative number of six cases since 2022.^36,49^

Sudan, on the other hand, is a country that is in close geographical proximity to the endemic regions, faces multiple crises, and experiences poor health settings and limited resources along with the presence of refugee populations. Mpox was first reported in October 2005 in a child and his mother from southern Sudan.^50,51^ From 2022 to 2024, 19 confirmed cases and one death were reported. However, this number could be underestimated due to the country’s unique circumstances.^36^

Overall, the situation in the MENA region, according to the WHO risk assessment, is moderate.^52^ However, the rapid escalation of Mpox from being an endemic problem in Africa to becoming a global health threat by 2022 indicates that the virus has the potential to spread more widely than expected and underscores significant weaknesses in research and public health responses. Moreover, the current number of cases, particularly in the MENA region, could be underestimated due to its perceived association with risky sexual practices, mainly according to evidence from the 2022 outbreak, which is still stigmatizing in many countries; thus, patients may not report mild and self-limited illness to the healthcare providers. Additionally, the ongoing conflicts in several countries in the MENA region have created conditions that might facilitate disease spread with the potential to evolve into a public health crisis. Overcrowding in refugee camps, poor health infrastructure, limited access to healthcare facilities, and the presence of other health challenges, such as malnutrition and trauma, all contribute to possible missing and underreporting of cases and create conditions conducive to disease transmission.

## PUBLIC HEALTH CHALLENGES AND PREVENTIVE ACTION

Since the declaration of the WHO of the ongoing spread of the MPXV as a PHEIC,^33^ the need for urgent action from countries worldwide has been emphasized. It is essential to prioritize the containment of the outbreak, exploring all possible strategies to protect vulnerable communities. Effective infection prevention and control programs are vital for managing the situation, beginning with a risk assessment to identify factors that contribute to disease spread and those at the highest risk.^53^

The current circumstances in MENA countries are highly variable. Countries face different circumstances that can either promote or hinder the spread of infection. Arab Gulf countries, for instance, have both risks and strengths. On one hand, they have the potential to spread due to high population densities mainly in urban areas, high rates of international travel, large trade interconnections, and a significant proportion of migrant worker populations. However, they also have good healthcare infrastructures, effective outbreak containment preparations, and availability of vaccines as announced by some of the health authorities in these countries.^54^ In contrast, other MENA countries have challenges such as inadequate healthcare systems, conflict and crisis situations, economic instability, high immigration rates, and widespread poverty. The challenge in these countries requires collaboration with international organizations to support public health response.

The following are suggested public health strategies for Mpox control and prevention:^24,54,55^

1. To effectively reduce the global spread of Mpox, it is essential to focus on controlling outbreaks in the affected African countries. Key actions include enhancing contact tracing, improving diagnostic tools, and expanding vaccination coverage in these regions.
2. Surveillance systems are crucial for the early detection and management of Mpox outbreaks. This includes monitoring suspected cases and high-risk individuals, along with efficient contact tracing.
3. Quarantine and isolation for suspected or confirmed Mpox cases until they are no longer infectious to others.
4. Treatment of the cases by antiviral medications such as tecovirimat can assist in decreasing viral replication thus minimizing infectivity to others.
5. Protection of healthcare professionals via wearing the appropriate personal protective equipment, such as gloves, gowns, and masks. Additionally, they must receive training on how to deal with suspected cases and improve case identification and management.
6. Increase public awareness through conducting campaigns. Engaging the community is crucial, and addressing the stigma associated with the disease is also important.
7. Vaccination is one of the main strategies to control Mpox outbreak. Expanding vaccination coverage is critical. The vaccine helps to lower infection rates, decrease infectivity, and decrease disease severity. Post-exposure prophylaxis and vaccinations for healthcare professionals and high-risk individuals should be prioritized.

The WHO approved the Modified Vaccinia Ankara-Bavarian Nordic (MVA-BN) vaccine as the first vaccine for Mpox in its prequalification list on September 2024.^56^ It is based on an attenuated strain of the Chorioallantois Vaccinia virus Ankara. MVA-BN vaccine has shown high immunogenicity as it induces both cellular and humoral immune responses.^57^ It is administered in two doses, 4 weeks apart, for individuals over 18 years. Though not licensed for children, pregnant women, or immunocompromised people, off-label use is permitted in outbreak situations. A single dose offers 76% effectiveness, while two doses provide 82% protection against the virus. Post-exposure vaccination is less effective. WHO is also evaluating other vaccines, including the Lister Clone-16 vaccine and the Acambis, Canton, Massachusetts2000 (ACAM2000) vaccine, for emergency use listing.^56^

## LIMITATIONS OF THE STUDY

There are several limitations to this review. First, there is no comprehensive information concerning the treatment outcome, vaccine availability, and vaccine effectiveness in the MENA region. Second, the study statistics primarily rely on secondary sources, such as the WHO reports and official announcements from governments, rather than original research with detailed epidemiological data. Although there is growing interest in Mpox research studies, several aspects regarding this disease are still not clear, and further studies need to be conducted to address the gap in our knowledge. Furthermore, there is a shortage of resources for the diagnosis and control of disease. Vaccines are still under investigation and limited coverage to areas in most need. Children, pregnant women, and immunocompromised individuals represent vulnerable groups, and there are no specific treatment guidelines for Mpox cases to date.

## CONCLUSION

The review provided a comprehensive understanding of Mpox, from its initial identification as a zoonotic disease to its emergence as a global health concern. There is limited published data on the spread of Mpox in the MENA region. The first case in the MENA region was detected in the 2022 outbreak. The number of detected cases has increased since then, but the cases in the MENA regions still represent less than 1% of global Mpox cases.

According to the WHO assessment, the Mpox risk is moderate in the MENA region. However, the rapid spread of the virus and the increase in the number of cases globally from 2022 to 2024 points toward the weaknesses in public health responses in facing the outbreak and the potential threat to all countries, including the MENA region.

The actual number of cases in the MENA region could be underestimated due to underreporting, stigma around sexual transmission, and ongoing conflicts in several countries. These countries also have several factors that facilitate disease transmission and contribute to the potential underreporting of cases, such as poor health infrastructure, limited access to healthcare facilities, malnutrition, overcrowding, and conflict. Collaborative research and resource allocation are essential for enhancing the preparedness and response capacity of this region.

## Acknowledgments

None.

## Authors’ contribution

The author of this manuscript was independently responsible for conceptualization, literature review, manuscript drafting, the final approval of this version, and ensuring the integrity of the information presented.

## Conflicts of interest

The authors declare no conflicts of interest.
